# Total En Bloc Spondylectomy in a Case of Solitary Metastatic Breast Carcinoma With Intact Neurology: A Case Report

**DOI:** 10.7759/cureus.67126

**Published:** 2024-08-18

**Authors:** Kiran K Mukhopadhyay, Ritwika Nandi, Aniruddha Sinha Sarkar, Ananda Mandal

**Affiliations:** 1 Orthopedic Surgery, Nil Ratan Sircar Medical College and Hospital, Kolkata, IND; 2 Orthopedics, Nil Ratan Sircar Medical College and Hospital, Kolkata, IND; 3 Orthopedics, Dr. P K Saha Hospital, Coachbehar, IND

**Keywords:** long follow up, intact neurology, breast carcinoma, solitary metastasis, en bloc spondylectomy

## Abstract

The management of spinal metastasis varies from patient to patient, depending on the type of lesion, stage of the disease, extension into the spinal canal, associated fractures, and life expectancy. We present a case of solitary metastasis with intact neurology in a 48-year-old lady who underwent a radical mastectomy for T2 N3 M0 breast carcinoma 34 months ago. Total en bloc spondylectomy in a neurologically intact patient is a challenging one. In all posterior approaches, there is a high chance of postoperative neurodeficiency. In our case, a combined approach seems to be a much safer procedure with easy accessibility to remove the total D8 vertebra.

## Introduction

Spinal metastasis accounts for 90% of spinal tumors encountered in spinal imaging [1]. The most commonly encountered site of metastasis is the dorsal spine, followed by the lumbar region [2]. The most common primary is found to be breast cancer in about one-fifth of cases [1]. In a cadaveric study, cases with primary breast and prostate cancer were examined to evaluate the prevalence of spinal metastasis. About 70% to 90% of cadavers were positive for metastasis.

The management varies from patient to patient, depending on the type of lesion, stage of the disease, extension into the spinal canal, associated fractures, and life expectancy. The NOMS framework, considering neurologic, oncologic, mechanical instability, and systemic disease, was proposed by Laufer et al. in 2013 to aid in decision-making [3]. The complex anatomy of the spine, major vessels, organs, and spinal cord all preclude the probability of a wide surgical margin in spinal tumors [4]. Surgeries aimed at reducing the tumor load would resort to curettage and piecemeal extraction, with ample exposure of the normal cells to tumor tissue in the process.

Total en-bloc spondylectomy was first introduced in primary malignant spinal tumors contained in the body as an alternative to intra-lesional piecemeal resection to achieve an adequate tumor-free margin with minimal risk of local spillage of oncogenic cells. Since then, variations have been implemented in various scenarios with good results. Any improvement in tumor-related mortality has to be balanced against the morbidity associated with the surgery for every tumor before any decision can be reached [5].

## Case presentation

**Figure 1:**
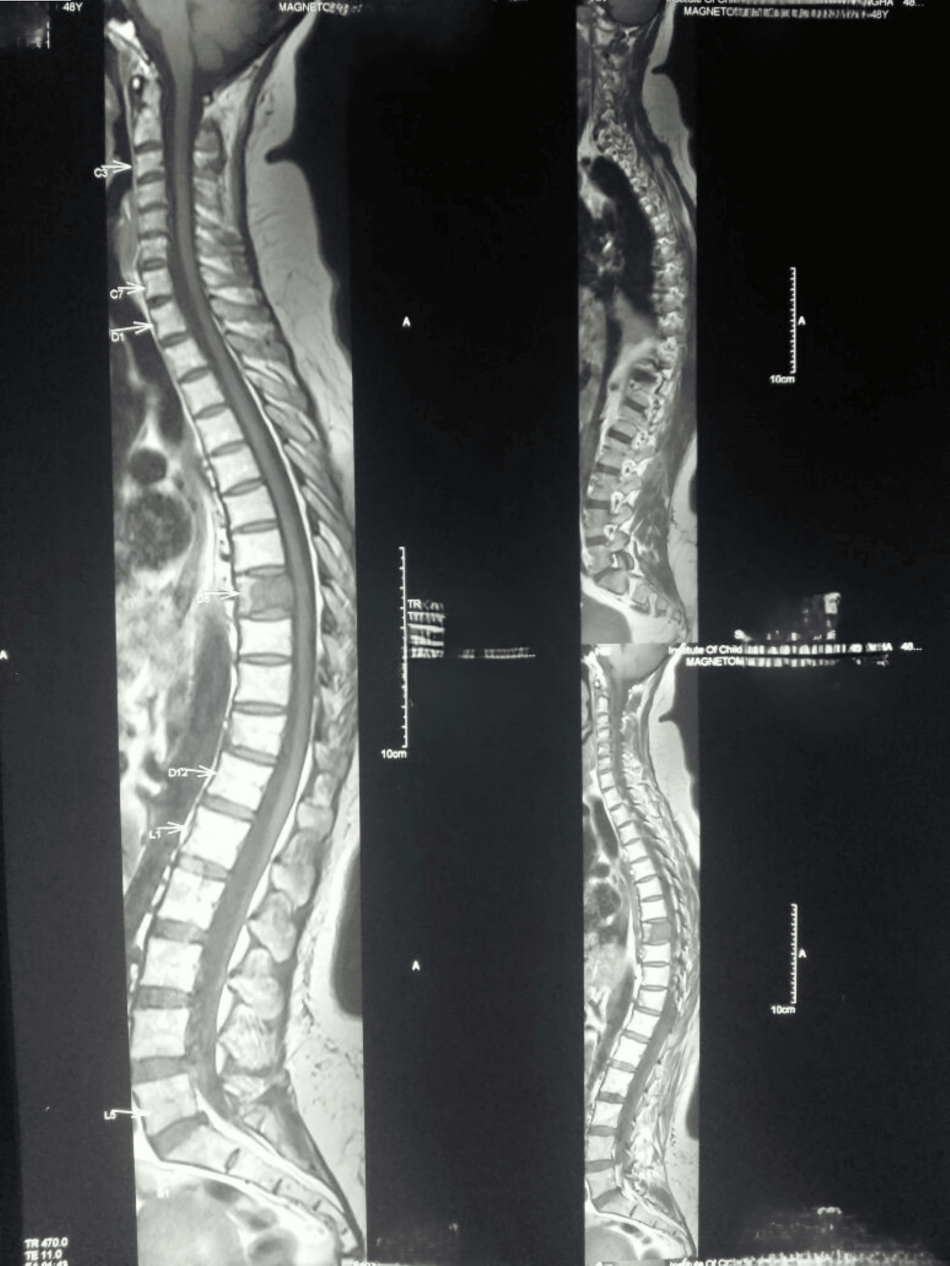
MRI showing solitary metastasis in D8 D8: eighth dorsal vertebra

A 48-year-old lady underwent a radical mastectomy for T2 N3 M0 breast carcinoma 34 months back, developed mid-dorsal pain, and was investigated. X-ray screening revealed nothing. MRI screening showed a lesion in the eighth dorsal vertebra (D8) (Figure 1). A PET CT scan was ordered which showed a lesion at D8 with increased uptake. D8 was the only area with metastasis seen in the MRI and PET CT scans.

After consultation with an oncologist, a total en-bloc spondylectomy of D8 was planned as it was a case of solitary metastasis. As our patient had a lesion in the mid-dorsal region (D8) with intact neurology, we planned to do a combined approach in a single sitting from back and front.

After the pre-operative workup and pre-anesthetic check-up, under general anesthesia, the patient was positioned prone on a radiolucent table. After proper dressing and draping, D8 was identified using an image intensifier, confirmed from both below and above. A posterior midline incision was made centering over D8. After paraspinal dissection an en bloc laminectomy was done and transpedicular resection was done up to D8 body. The pedicle screw was inserted at D6 and D10, and fixed with rods. The posterior incision was closed after putting a suction drain.

**Figure 2:**
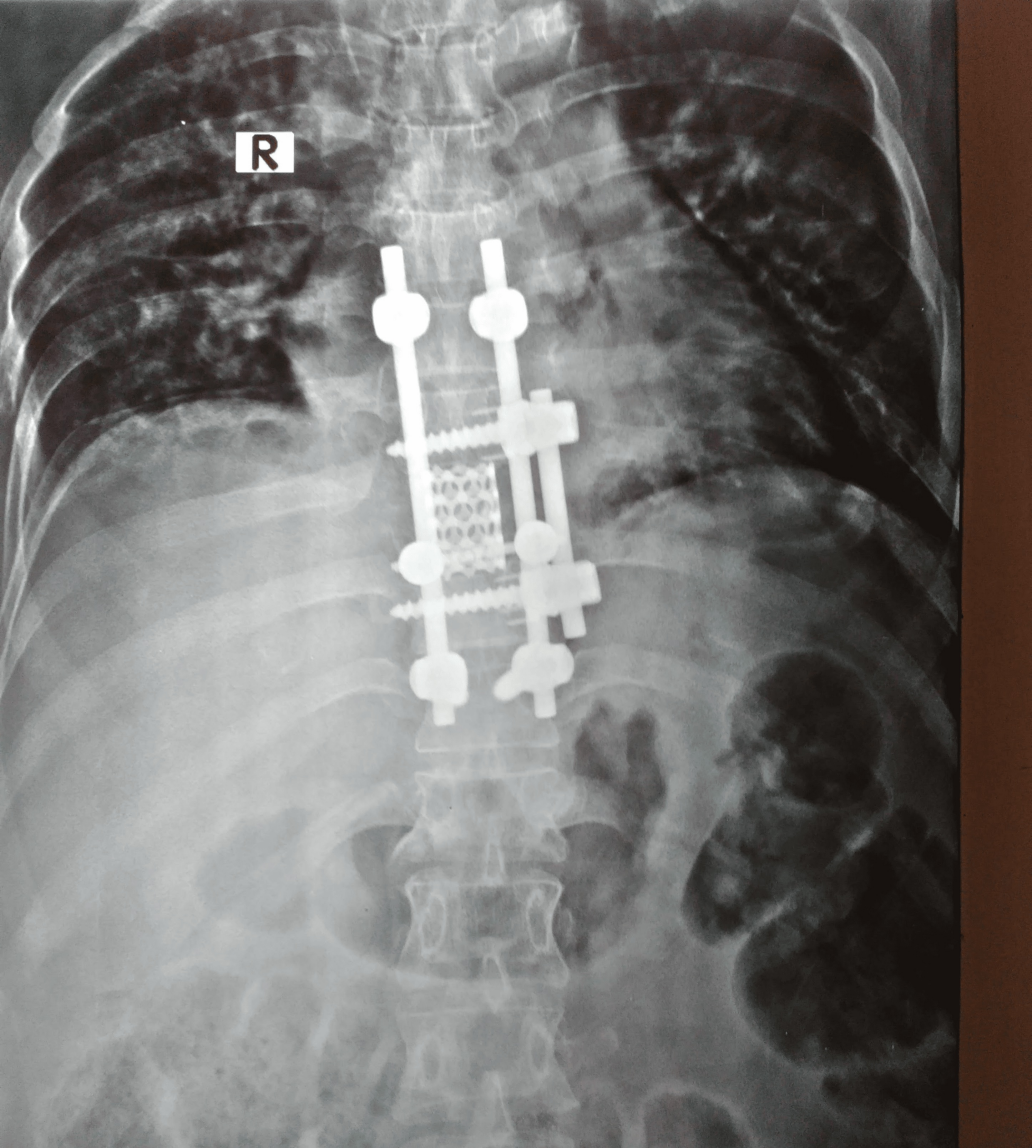
Postoperative X-ray AP view

**Figure 3:**
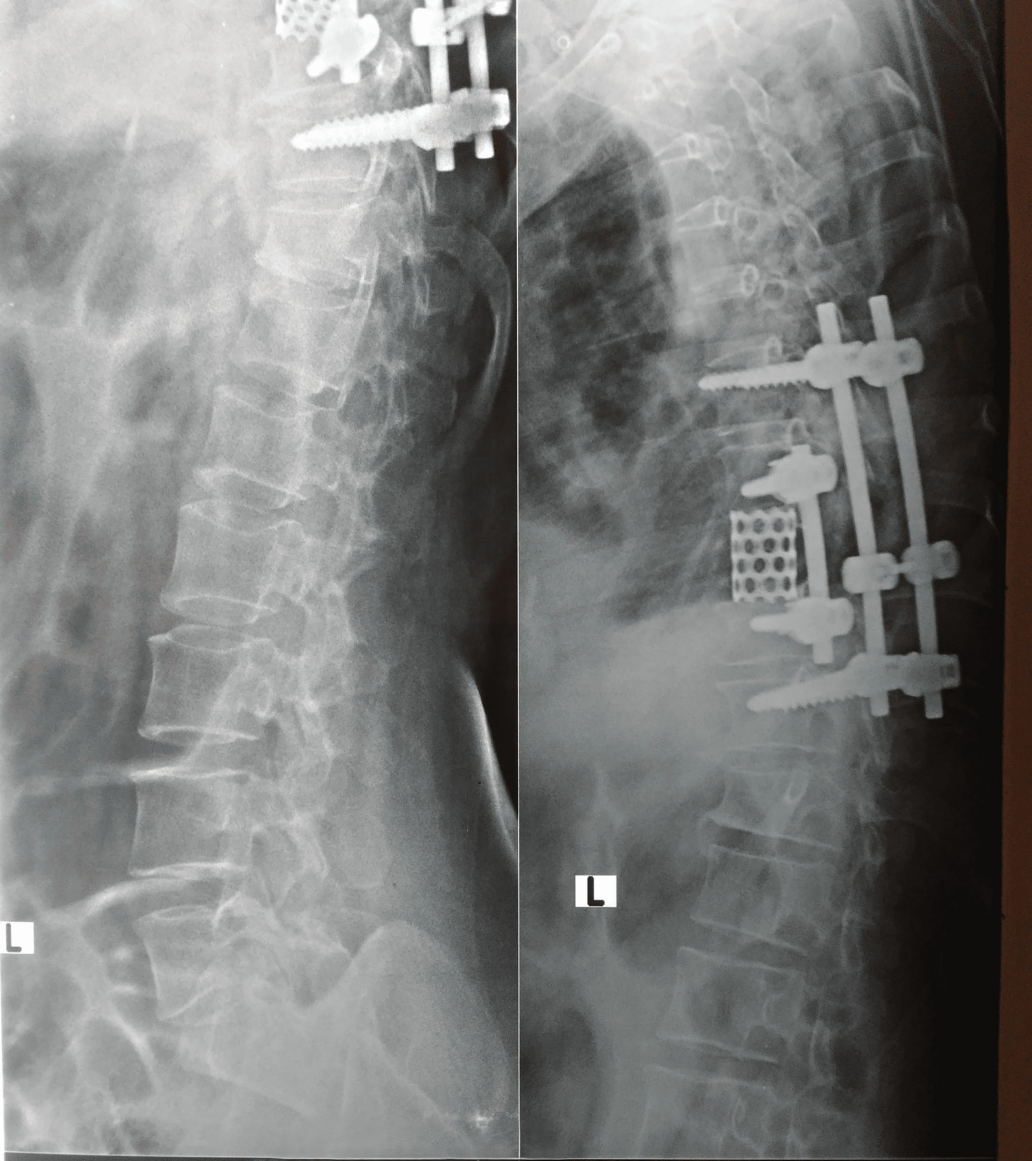
Postoperative X-ray lateral view

The patient was then turned and positioned laterally with the left side up over a kidney bridge. An incision was made over the left sixth rib. After exposure, segmental arteries were ligated and D8 was completely removed with adjacent discs. A staple washer was placed over D7 and D9 bodies and the void of D8 was filled up with a titanium mesh cage filled with autogenous bone graft. Bicortical pedicle screws were inserted through staple washers and fixed with an appropriately contoured rod. Over a number 28 chest drain, the wound was closed. Postoperatively, the patient was shifted to the intensive coronary care unit (ICCU) for two days and after the removal of the chest drain and suction drain and after obtaining a satisfactory postoperative X-ray (Figures 2, 3), the patient was mobilized with a hyperextension brace.

Postoperatively, the patient was neurologically intact and healed satisfactorily.

We followed up with the patient regularly for one month, then every quarter. A one-year CT scan showed satisfactory results.

No recurrence of symptoms was reported for six years postoperatively. The patient was regularly followed up by an index surgeon, had metastasis after six years to other soft tissues, and expired after treatment at six years and six months.

## Discussion

The present case study entails that of a patient without any neurological improvement and with a good oncological prognosis. Hence, the solitary tumor was managed with a single-level spondylectomy.

Tomita et al. [4] coined the term “total en bloc spondylectomy” or vertebrectomy in 1997. Seven patients with spinal tumors were managed with this aggressive surgery and reported satisfactory clinical and oncological outcomes. Most patients reported improved neurological status by at least one grade after the procedure. One patient succumbed, which was attributed to mediastinal metastasis. En-bloc resection of the posterior elements was removed, followed by anterior column resection.

Magerl and Coscia [6] preferred the posterior approach for a total vertebrectomy in nine cases. They concluded that the neural decompression achieved was adequate via this method as well. Howell et al. [5] advocated for the technique as long as proper patient selection and necessary surgical skills were available. Local recurrence was reported to be higher in patients' post-radiotherapy, intra-operative dural tears, and tumors occupying more than half the neural canal.

Igarashi et al. [7] evaluated 91 patients retrospectively and reported 11% local recurrence, with prior radiotherapy as a significant risk factor. However, whether the low local recurrence rate reflected a longer overall survival was unclear.

Park et al. [8] studied 32 patients with spondylectomy to evaluate any implant-related complications, and they reported that 12 of those patients demonstrated rod fractures within five years of the index surgery. Satellite rod techniques, dual constructs, and multi-rod constructs have all been suggested to decrease pseudoarthrosis and fatigue fractures at the osteotomy site [9,10].

Ohashi et al. [11] performed surgery on 18 patients with spinal metastasis and concluded that, although the surgical margins achieved were adequate in the index surgery, 70% of patients developed distant metastasis, with a median time of 21 months from the spine surgery.

The complications of this surgery include possible tumor spillage, neuronal damage, and major vessel injury, particularly during the blunt dissection of the anterior body. Hypotensive anesthesia, pre-operative embolization, careful dissection, etc. have been recommended to minimize the risk of excessive bleeding [12].

Yao et al. [13] concluded that for oligometastatic solitary and spinal metastases with biologically favorable histological findings, en bloc spondylectomy was the treatment of choice.

## Conclusions

Total en bloc spondylectomy in a neurologically intact patient is a challenging one. In all posterior approaches, there is a high chance of postoperative neurodeficiency. In our case, a combined approach seems to be a much safer procedure with easy accessibility to remove the total D8 vertebra.
